# COVID-19 lockdown may increase cardiovascular disease risk factors

**DOI:** 10.1186/s43044-020-00127-4

**Published:** 2021-01-05

**Authors:** Daha Garba Muhammad, Ibrahim Ahmad Abubakar

**Affiliations:** Department of Physiotherapy, Abubakar Tafawa Balewa University Teaching Hospital, Bauchi, Nigeria

**Keywords:** CVDs, COVID-19, Physical activity, Tobacco, Alcohol

## Abstract

**Background:**

Coronavirus disease 2019 (COVID-19) is a disease caused by severe acute respiratory syndrome coronavirus 2 (SARS CoV-2) and was declared a worldwide pandemic by the World Health Organization (WHO) on 11 March 2020 which is leading to significant morbidity and mortality. In compliance with WHO recommendation of movement restrictions, many countries have imposed compulsory self-quarantine and restricted movements of their citizenries (lockdown/sit at home) and closure of businesses and borders as preventive measures to the fast-spreading virus. Consequently, this decision has made the emergence of behaviors that are detrimental to cardiovascular diseases (CVDs) which are the leading cause of the global mortality rate.

**Main body:**

The increase in sedentary lifestyles, alcohol consumption, and substance abuse during COVID-19 pandemic lockdown as a result of personal restrictions in COVID-19 lockdown is linked with the risk of death from chronic diseases such as cardiovascular diseases (CVDs).

**Conclusion:**

The lockdown has increased risk factors of CVDs, and as such, there might be an increase in the number of non-communicable disease (NCD)-related mortality rate. The effect does not end during the period of coronavirus pandemic but even after the pandemic.

## Background

Coronavirus disease 2019 (COVID-19) is a disease caused by severe acute respiratory syndrome coronavirus 2 (SARS CoV-2). The viral outbreak was first discovered in Wuhan city of Hubei province of China in December 2019 [1]. It was declared a worldwide pandemic by the World Health Organization (WHO) on 11 March 2020 which is leading to significant morbidity and mortality [2]. With an escalating number of governments hardening nationwide quarantine, many countries have imposed compulsory self-quarantine and restricted movements of their citizenries (lockdown/sit at home) and closure of businesses and borders as preventive measures [2, 3].

Cardiovascular diseases (CVDs) are a group of diseases that affect the heart and blood vessels such as the coronary arteries, stroke, and heart attacks [4]. CVDs are the number one cause of global deaths [4]. As COVID-19-related deaths are said to be high in persons with an underlying comorbidities such as CVDs, several studies had established a link between coronavirus deaths and CVDs [5] with little attention paid to the impact of the coronavirus consequences on the emergence of new cases of CVDs. Cardiovascular disease risk factors are usually classified into modifiable and non-modifiable. The modifiable ones include a healthy lifestyle while non-modifiable ones are age, gender, race, and hereditary. CVDs are preventable through modifying the modifiable risk factors which are the eating habits, physical activity, tobacco usage, and harmful use of alcohol [4]. However, COVID-19 lockdown enforced by the governments of different countries to curb further the spread of this virus has affected the lifestyle of people which consequently favors some habits that expose an individual to the risk of CVDs. These habits may also heighten the risk of CVDs in persons at high risk of CVDs such as older adults or genetically exposed individuals. In 2013, a global action for the prevention and control of NCDs was launched with the aim of reducing NCD-related premature deaths by 25% by 2025 [4]. Due to the burden of the CVD mortality rate, two of the set targets were directed towards prevention and control of CVDs [4]. Achieving this goal might be affected in this situation of lockdown since the lockdown promotes an increase in sedentary lifestyle and other CVD-related risk factors.

## Main body

Physical activity (PA) is defined as any bodily movement produced by the skeletal muscles that requires energy expenditure above resting levels, in addition to heart rate and breathing frequency [6]. The risk of death from chronic disease such as CVDs is generally the highest among the least fit and physically inactive and the lowest among the most fit and physically active [7]. Physical inactivity as a result of personal restrictions in the COVID-19 lockdown era may hamper physical activity prophylaxis effects on CVDs [8]. It was already established that there exists an inverse relationship between leisure time physical activity and the risk of cardiovascular mortality regardless of age, sex, and the presence or lack of pre-existing cardiovascular disease [9].

COVID-19 lockdown may increase the risk of CVDs by promoting an increase in unhealthy eating behavior and decrease in physical activity [10]. Improper eating habit due to the lack of healthy food availability may result in weight gain which in turn can cause obesity and lipid accumulation in the blood vessels [11]. Increased social isolation, loneliness, boredom, anxiety, and depression generated by the pandemic might have played major roles in the lifestyle changes, emotional changes, and mood disorders which may influence food choices, with the search for comfort foods, such as processed snacks and sweets [12, 13]. Increased consumption of sweetened beverages, including soft drinks, cordial (a sweet, flavored, concentrated syrup that is mixed with water to taste), and fruit juices, may contribute to the development of obesity [14].

Lockdown is a complex social phenomenon which may provoke many behavioral responses, and alcohol use has been shown to be associated with stress [15]. Thus, the period of isolation may trigger an increase in alcohol misusage, relapsing, and emergence of new alcohol misusers [15]. An interplay among lockdown-related consequences such as the increase in financial problems, loneliness, and uncertainty about the future may increase the rate of alcohol consumption during lockdown period of COVID-19. Twenty-four percent (24%) of participants in a particular study on the effects of alcohol consumption among patients with pre-existing alcohol use disorder increase their alcohol consumption rate while 19% decrease their rate of consumption [16]. It was also reported that 38% of the respondent abstained from alcohol consumption before lockdown, but 17% of the respondents that abstained from drinking before lockdown had relapsed to alcohol consumption [16]. Only 12% of the participants drinking alcohol abstain from the act during lockdown [16]. A study by Chodkiewicz et al. [17] found a 30% change in the pattern of their alcohol consumption among the participants in the study, with 16% increasing their rate of alcohol consumption and 14% reducing their consumption habit. Furthermore, a 240% rise in alcohol online sales was reported [18]. It was also stated that 1 in every four (25%) of alcohol consumers drink alcohol more frequently since the beginning of COVID-19 lockdown [19]. The increase in drinking rate may be due to boredom and having more free time [19] due to the stay-at-home nature of the lockdown.

Tobacco usage and increased risk of CVDs have since been established [4]. Tobacco usage is the second substance being abused during COVID-19 lockdown [17]. An increase in tobacco usage was also noted in Italy, India, South Africa, the UK, and the USA during the COVID-19 lockdown [20]. The effect of tobacco smoking does not only affect the smoker but a family member exposed to the second-hand smoke [21]. Second-hand smoking also increase one to a CVD risk factor by 30% [22].

## Conclusion

It was evidenced that COVID-19 lockdown has the potentials of increasing CVDs and its mortality rate by indirectly increasing CVD risk factors such as physical inactivity, improper eating habit, tobacco usage, and alcohol misusage. This in turn may escalate CVD-related premature deaths which can reduce the success of 2013, global action for the prevention and control of NCD premature deaths by 25% by 2025. However, no much efforts were put to study the effects of the COVID-19 lockdown on CVD risk factors, rather the studies focus mainly on the effect of the CVDs on COVID-19 complication and mortality. Therefore, proper awareness on social media, radio, and other means of mass communication on living a healthy lifestyle will do much in reducing the problem. In addition, a systematic review or meta-analysis should be conducted to compare CVDs and their risk factors during the pre-COVID-19 era and during the pandemic.

## Data Availability

Not applicable

## Abbreviations

CVDs: Cardiovascular diseases
NCDs: Non-communicable diseases
WHO: World Health Organization
PA: Physical activity
